# American Medical Association at Portland

**Published:** 1904-12

**Authors:** 


**A Monthly Review of Medicine and Surgery.**

*EDITOR:*

WILLIAM WARREN POTTER, M. D.

All communications, whether of a literary or business nature, books for review and exchanges, should be addressed to the editor: 284 FRANKLIN ST., BUFFALO, N. Y.

VOL. XLIV.—LX. DECEMBER, 1904. No. 5

### American Medical Association at Portland.

IT HAS been announced already that the American Medical Association will meet at Portland, Oregon, July 11-14, 1905. For the third time in its history the association has sought the Pacific coast region to hold its annual meeting, first, in 1876; second, in 1894, each time in San Francisco; and now for the third excursion to the Occident Portland is selected as the city of the convention.

In order to bring the officers of the sections into closer touch, the president-elect, Dr. Lewis S. McMurtry, of Louisville, called them in conference at the new Hotel Astor, New York, on Monday evening, November 14, 1904. Around a dinner table of faultless service were gathered representatives from Atlanta, Baltimore, Boston, Chicago, Louisville, Philadelphia, New York, and other cities, who discussed the various problems relating to the scientific and business interests of the association, with a particular view to ensure the success of the meeting at Portland.

Dr. George H. Simmons, the indefatigable secretary and the editor of the association journal, presented in considerable detail the duties of the officers of sections under the present by-laws and regulations, and gave interesting information concerning pending negotiations for transportation. After he had concluded, many questions were propounded by the section officers, all of which received prompt answer, and when the last word had been said by the secretary, the impression was very distinctly left that he understood the duties of his office and was performing them with conscientious and conservative regard for the interests of every member of the association.

In the course of the conference it was developed that many of the sections already had made considerable progress in the preparation of their programs, while all were well under way and will be easily completed before needed by the printer. One of the rules of the association, and an excellent one, is that no paper shall be placed upon the program, the author of which does not furnish an abstract. If this were the regulation in all state and national medical societies it would be well. How can an author expect a good discussion unless an advance publication of an abstract of his paper is made? No man can make a proper abstract of a paper, except the writer of it. He knows its shuck and kernel, and he only can separate them adequately for mental digestion and assimilation.

It transpired at this conference that transportation lines are manifesting already a disposition to grant liberal rates across the continent. It seems quite probable from present indications that not more than one fare for the round trip will be demanded, and possibly a rate a trifle less than that will be agreed upon. Furthermore, all who pay the rate will be granted the privilege to go by one route and return by another, and in addition liberal stopover facilities will be conceded. This will enable tourists to visit all important localities west of the Rocky Mountains.

Dr. Joseph D. Bryant, chairman of the committee on national incorporation of the association, by invitation of the president, entertained the guests with an interesting account of the progress making by his committee, from which it appeared that ultimate success is not far distant.

The sum total accomplished at this meeting was all that could be desired and the value of such conferences cannot be overestimated. It enables the officers to understand each other and to establish harmonious relations long before they meet for scientific work at the convention. Thus they methodise and concentrate energy, forging from heterogeneous elements a compact instrument that accomplishes a result not otherwise to be obtained. That the Portland meeting will be a success seems already assured.

---

ACCORDING to a Saint Louis despatch to *The Tribune* of November 24, 1904, a specialist in the World's Fair city removed a grain of rice from the ear of a New York bride, who had suffered untold tortures for several days, because of the presence of this foreign body. It was the untoward result of showers of rice thrown at the young couple as they departed on their wedding journey, one grain of which made lodgment in the young woman's ear. At Niagara Falls next day the trouble began, the swelling of the rice in the canal causing intense pain, and finally at Saint Louis the bride of three days fainted. Though the cause is removed, the effects are yet serious. It is not impossible for such a condition to result in permanent deafness in the ear involved. This incident,—this grave accident,—should prove a warning to young people to cease the barbarous practice, not only of rice throwing, but of other equally abominable customs at weddings.

---

CALOX, the new dentifrice mentioned in these columns last May, has already taken its place in the foreground of preparations intended for cleansing the mouth and the preservation of the teeth. It is chemically perfect, scientifically accurate, and hygienically absolute. No refined person having once used it, will consent to be without it. Every physician and dentist of repute or standing once familiar with it will recommend it to their patients. It represents the most decided advance in mouth hygiene and tooth preservation that has been made within recent years.

---

A MOVEMENT has been started throughout the lower east side of New York city to educate the people up to the point of keeping the streets clean.

At the university settlement it is said all the Jewish rabbis will be asked to address their congregations on the subject, while at the same time the principals and teachers in the public schools will proceed along the same lines by delivering short talks on the hygienic benefits of having clean streets, and by appealing to the pupils in their schools to teach their parents how to keep the streets free from dirt.

